# Perceptions and Experiences of Primary Care Providers on Their Role in Tobacco Treatment Delivery Based on Their Smoking Status: A Qualitative Study

**DOI:** 10.3390/healthcare12242500

**Published:** 2024-12-11

**Authors:** Stavros Stafylidis, Sophia Papadakis, Dimitris Papamichail, Christos Lionis, Emmanouil Smyrnakis

**Affiliations:** 1 Laboratory of Primary Health Care, General Practice and Health Services Research, School of Medicine, Aristotle University of Thessaloniki, 541 24 Thessaloniki, Greece; smyrnak@auth.gr; 2 Clinic of Social and Family Medicine, School of Medicine, University of Crete, 710 03 Heraklion, Greece; sophia@ncsct.co.uk (S.P.); lionis@uoc.gr (C.L.); 3 Department of Public Health Policy, University of West Attica, 12243 Attica, Greece; dpapamichail@uniwa.gr

**Keywords:** primary care providers, smoking status, barriers, facilitators

## Abstract

Introduction: Despite the well-documented benefits of smoking cessation interventions, the implementation and success of these programs in primary care settings often encounter significant barriers. A primary care provider’s personal smoking status has been identified as a potential barrier to tobacco treatment delivery. The aim of this qualitative study is to explore the experiences and perspectives of primary care providers regarding their role in delivering smoking cessation interventions to patients based on their personal smoking status. Specifically, the study seeks to examine providers’ thoughts, emotions, and behaviors concerning their own smoking behavior and to understand their attitudes and actions when supporting patients who smoke and to explore their perspectives on the effectiveness of training programs designed to promote tobacco treatment. Materials and Methods: Semi-structured interviews were conducted with 22 primary care providers from six public primary care units in the Central Macedonia Region, Greece. Thematic analysis was used to analyze data. Results: Healthcare providers who are current smokers may face unique challenges in effectively counseling patients on smoking cessation. On the contrast, non-smoking and especially previous smoking healthcare providers were noted to exhibit greater confidence and efficacy in delivering cessation support, often serving as role models for patients aiming to quit smoking. Participating in structured cessation training programs often led healthcare professionals to reflect and reevaluate their own smoking behaviors. Conclusions: Personal smoking status of primary care providers impacts the delivery of tobacco treatment, affecting their credibility and effectiveness in providing cessation support. Educational programs positively impact attitudes and behaviors, underscoring their importance in improving both PCPs’ professional effectiveness and personal health outcomes. These findings suggest that addressing PCPs’ smoking habits and enhancing training opportunities are critical for optimizing smoking cessation services.

## 1. Introduction

### 1.1. Background

Tobacco treatment delivery is a gold standard public health intervention and key to reducing the burden of smoking-related diseases, which include cardiovascular disease, chronic respiratory conditions, and various cancers [1,2]. Primary care providers (PCPs) including General Practitioners (GPs), nurses and health visitors, play a pivotal role in these efforts due to their frequent interactions with patients and their position to offer repeated and personalized advice on quitting smoking, but face challenges such as limited resources and inadequate training [3]. Health visitors are also instrumental in reaching out to smokers, providing counseling, and facilitating access to cessation resources [4]. PCPs’ smoking status or other addictions significantly impact their clinical practices, particularly concerning smoking cessation interventions. Research indicates that healthcare providers who smoke are less likely to engage in tobacco treatment counseling and may feel less confident in their ability to influence patients’ smoking behaviors due to perceived hypocrisy or personal struggles with addiction [5]. Conversely, non-smoking PCPs or those who have successfully quit smoking tend to be more proactive in initiating conversations about tobacco treatment and providing support to their patients [6].

In Greece, despite the high smoking prevalence among healthcare professionals, which has been reported to reach 45% in some studies, the country remains one of the EU members with the lowest investment in tobacco control and cessation programs [7]. In southern European countries like Greece, where cultural and social norms heavily reinforce smoking behaviors, healthcare professionals are similarly affected by these facts [8]. This socio-cultural environment contributes to the normalization of smoking even among those tasked with promoting health, further underscoring the challenges faced in implementing effective cessation interventions. Greece also lags behind many European nations in implementing policy-driven measures such as smoke-free public areas and public education campaigns aimed at reducing the normalization of smoking [9,10]. In more depth, smoking cessation services are shaped by disparities in healthcare access, cultural norms, and the role of primary care providers. Urban areas like Athens and Thessaloniki benefit from specialized healthcare services, while rural regions rely on primary care, often lacking comprehensive cessation resources [11]. The cultural acceptance of smoking and low risk awareness further hinder cessation efforts, compounded by inconsistent enforcement of public health policies [9]. Educational programs aimed at improving the skills of primary care providers in tobacco treatment are essential, yet they are not uniformly implemented across the country [12]. These programs in a Greek setting include structured training on brief interventions, counseling, and evidence-based strategies such as motivational interviewing and integrating cessation advice into clinical practice. The training programs are delivered through workshops, online modules, and interprofessional team training, with durations ranging from single-day sessions to ongoing initiatives. Participation is generally encouraged but not always mandatory [11].

Despite the well-documented benefits of tobacco treatment interventions, the implementation and success of these programs in primary care settings often encounter significant barriers [13]. These barriers can range from a lack of time during consultations and insufficient training in tobacco treatment techniques, to perceived patient resistance and inadequate organizational support [14]. There are also numerous facilitators that can enhance the effectiveness of tobacco treatment interventions by primary care providers. These facilitators include comprehensive training programs, the integration of tobacco treatment guidelines into clinical practice, the use of evidence-based intervention tools, and the support from healthcare organizations [15,16]. Exploring and understanding these barriers and facilitators related with PCPs’ smoking behavior is essential for developing strategies to optimize tobacco treatment delivery efforts in primary care settings [17].

### 1.2. Aim

The aim of this qualitative study is to explore the experiences and perspectives of primary care providers regarding their role in delivering tobacco treatment interventions to patients, with a particular focus on how their personal smoking status influences their approach. Specifically, the study seeks to: (a) examine providers’ thoughts, emotions, and behaviors concerning their own smoking behavior; (b) understand their attitudes and actions when supporting patients who smoke; and (c) explore their perspectives on the effectiveness of training programs designed to promote tobacco treatment. The insights generated from this study may inform the development of more effective support systems for both primary care providers and patients [18].

## 2. Materials and Methods

### 2.1. Study Design

A qualitative study was undertaken involving semi-structured interviews with primary care providers in the Central Macedonia Region, Greece. The research was carried out from June to September 2023. Thematic analysis was employed to identify and analyze patterns within the data. The consolidated criteria for reporting qualitative research (COREQ) were used to ensure comprehensive and transparent reporting of the study findings. This approach ensured that the study adhered to high standards of qualitative research methodology, enhancing the transparency, credibility, and reliability of the results [19].

### 2.2. Setting and Participants

The study aimed to capture a broad range of perspectives from various PCPs in Central Macedonia, Greece. To achieve this, a purposeful sampling method was employed to select a sample of participants from various primary health care centers [20]. The participants included physicians, nurses, and health visitors, ensuring representation from different professional backgrounds. To enhance the diversity of perspectives, the study included both smokers, non-smokers, and former smokers among the participants. Participants were initially approached via telephone, where the scope of the study was explained, followed by face-to-face visits for further engagement. Particularly, participants were informed about the study’s aim to explore the role of primary care providers in tobacco treatment delivery, focusing on how their smoking behavior influences their approach. They were also briefed on the interview process, the study’s benefits for smoking cessation in primary care, and assured of confidentiality and voluntary participation. Following the initial contact, a single face-to-face visit was arranged with each participant, to provide more details of the study and to address any revealed questions or concerns regarding their participation. All participants who were approached agreed to participate in the study and there were no withdrawals from the study. This selection process was guided by the maximum variation criterion, which focuses on information-rich cases capable of shedding light on the research questions, whereas the scope was to ensure that the sample represented a wide range of experiences and viewpoints, which could provide deeper insights into the research questions related to smoking cessation practices in primary care [20].

Participants were eligible for the study if they met the following criteria:

They were 18 years of age or older;

They could read and/or understand Greek;

They were currently working in a primary health care unit;

They were involved in working with smokers on a daily basis.

The sample size was determined based on the principle of saturation, a common practice in qualitative research, which ensures that no new information or themes emerge from the data. This approach guarantees that the collected data adequately reflects the variety of experiences and perspectives related to the research questions [21].

### 2.3. Theoretical Framework

This study is based on the Theory of Planned behavior (TPB) and the COM-B model. The TPB suggests that behavior is shaped by attitudes, subjective norms, and perceived behavioral control, which influence an individual’s intention to act. In this study, we examine how primary care providers’ smoking status and attitudes affect their role in delivering tobacco treatment interventions [22]. The COM-B model (Capability, Opportunity, Motivation—behavior) complements this by exploring how these factors enable or hinder PCPs’ behavior in supporting tobacco treatment delivery. By integrating both frameworks, we aim to understand the key influences on PCPs’ engagement in tobacco treatment based on their smoking status [23].

### 2.4. Procedures

The research team developed a comprehensive interview guide that included a series of open-ended questions designed to explore the experiences of the participants, along with follow-up probes to elicit more in-depth responses. It included questions on demographic characteristics, smoking history, reasons for smoking or quitting, and how their role as healthcare professionals influenced these behaviors and their clinical approach to smoking cessation. Additionally, the guide addressed the impact of smoking cessation programs on participants’ habits and professional practices, as well as barriers and motivators to quitting, providing a comprehensive framework for understanding the interplay between personal and professional factors. This guide was informed by existing literature [24] and expert input to ensure relevance and comprehensiveness. Before full implementation, the interview guide was pilot tested with two participants to assess clarity, flow, and the appropriateness of the questions. Feedback from the pilot participants was used to refine and improve the guide. Interviews were conducted between June and September 2023 by two experienced qualitative researchers (SS, ES), and had a mean duration of 35 min. The interviews took place in the participants’ healthcare facilities in-person, were audio-recorded with the consent of the participants, and field notes were taken by the interviewers to capture non-verbal cues and contextual details. The audio recordings were transcribed verbatim. The interview transcripts underwent a thorough quality check, with the researchers reviewing and correcting any transcription errors, including language and grammatical inaccuracies, to ensure the data accurately reflected participants’ responses. No repeated interviews were conducted, nor were transcripts returned to participants for review or correction. Participants did not have an existing relationship with the researchers and no other individuals were present during the interview.

*a*. *Data analysis*

The processing method utilized for analyzing the collected data through interviews was thematic analysis. According to Braun and Clarke (2006), “thematic analysis involves the systematic identification, organization, and understanding of recurring patterns of meaning within a dataset”. Through this process, the researcher detects and focuses on meanings relevant to their study, providing answers to their research questions [25]. Initial coding was developed based on interview questions and existing literature on related themes. Coding was made independently by two researchers (SS, ES) who also conducted the interviews. Regular team meetings were held to discuss coding strategies, data interpretation, the evolution of the coding framework, and the identification of key themes. The analysis involved iterative cycles of deductively and inductively derived coding. Differences in coding and theme identification were resolved through regular discussions and consensus meetings among the research team members. In cases of disagreement, the team reviewed the relevant data together, discussed alternative interpretations, and reached a mutual agreement to ensure consistency and reliability in the coding process.

To ensure the credibility and stability of the findings, the method of triangulation was used. In quantitative research terms, credibility reflects internal validity, corresponding to qualitative studies, and involves the identification, theoretical definition, and structuring of subcategories, categories, concepts, and conceptual constructs of the phenomenon under study. Stability refers to various aspects of data reliability and, by extension, the study’s results. Triangulation involves using various methods within a study to confirm or achieve completeness in understanding a phenomenon [26]. In this study, researcher triangulation was ensured by the involvement of three different researchers (SS, ES, SP), two of whom have extensive experience in qualitative research methodology (ES, SP). These researchers, along with the principal investigator (SS), were involved in the coding and analysis of the interview data.

## 3. Results

Study participants included 22 PCPs from Central Macedonia, Greece, who were employed in urban and semi-urban primary care centers of the local Public Health System. Demographic and professional characteristics are detailed in Table 1. 

**Figure 1:**
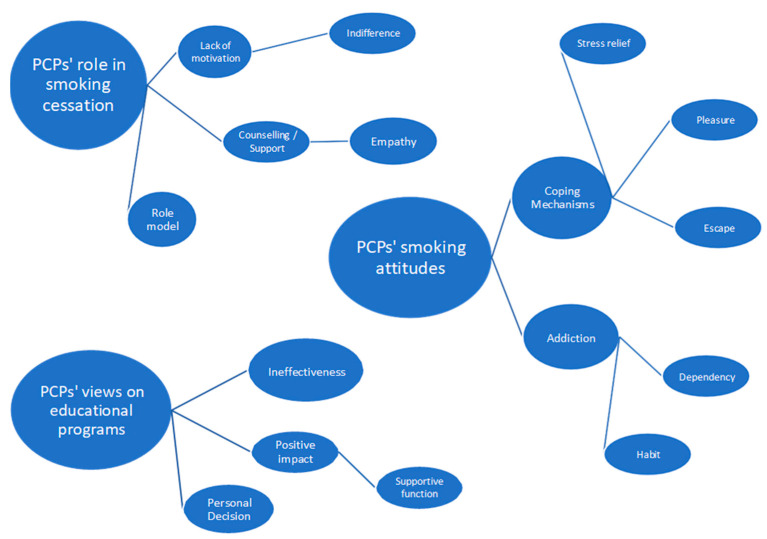
Thematic map of PCPs’ attitudes on personal smoking, their role in smoking cessation and their views on smoking cessation educational programs.

PCPs’ perspectives on smoking are explored through three main objectives: (a) their thoughts, emotions, and behaviors regarding their own smoking habits, (b) their attitudes and actions in supporting patients who smoke, and (c) their views on the effectiveness of educational programs on smoking cessation. Thematic analysis generated the thematic map presented in Figure 1.

***a***. ***PCP’s thoughts, emotions, and behaviors concerning their own smoking behavior.***

This section describes how smokers and former smokers perceive and justify their personal smoking behaviors. The thematic analysis of this part revealed three main themes regarding the thoughts, emotions, and behaviors of healthcare professionals concerning their own smoking behaviors. The themes included coping mechanisms such as stress relief, pleasure, and escapism, as well as considerations of addiction encompassing dependency and habitual behaviors.

**Coping mechanisms**

This theme includes the sub-themes of stress relief, pleasure, and escape. Respondents (smokers and former smokers) indicated that smoking helps them manage stress, derive pleasure, and escape from problems.

1. Stress Relief

Many healthcare professionals reported that smoking provides significant stress relief, helping them cope with their demanding jobs.

[...] “I feel that it helps, that I want it, especially after a difficult day in work it relieves me...”. GP, 30 years old, smoker.

[...] “Smoking two cigarettes at that moment (during stressful workload) helps me more. It takes away my stress. It helps me”. RN, 35 years old, smoker.

2. Pleasure

Smoking is also seen as a source of pleasure, offering a moment of enjoyment during their busy routines.

[...] “It is very pleasant as a habit, at least for me it was pleasant..I liked smoking and I remember that (smoking) was a happy moment during my stressful work”. RN, 40 years old, former smoker.

3. Escape

For some, smoking serves as an escape from their problems and provides a necessary break after strenuous tasks.

[...] “The reason I smoked was to escape from the problem that was bothering me. I was also utilizing it as a chance to get away from stressful tasks. It was something common among smokers colleagues” HV, 54 years, former smoker.

[...] “I also used it as a break. When you had a difficult task, you would say, ‘let me smoke a cigarette first to relax and then start’ or when you had to do some physical work, you would say, ‘okay, I’ll do it later’” GP, 40 years old, former smoker.

**Addiction**

The sub-themes of tobacco dependency and habitual behaviors emerged under this category. Smokers and former smokers PCPs described their smoking habits as highly addictive and deeply ingrained in their daily routines.

1. Tobacco dependence

Healthcare professionals likened smoking to drug addiction, emphasizing its powerful hold over them.

[...] “It’s like a drug. It is a drug, not just like one, it is a drug. And that is the main reason that is so addictive” RN, 42 years old, smoker.

[...] “I consider cigarettes to be... yes, a drug. It’s a drug that provides pleasure.. And then is like a prison and you cannot escape”. GP, 53 years old, smoker.

2. Habit

Smoking is described as a habitual act, that has become part of their daily routines and activities.

[...] “The daily habit... the habit, I believe Because I am smoking during certain circumstances in my daily life. For example, I cannot drink a coffee without smoking a cigarette”. RN, 55 years old, smoker.

[...] “and the routine, where you do the same things, like getting up in the morning, eating, having your cigarette. It’s part of your routine …”. RN, 40 years old, former smoker.

***b***. ***PCP’s attitudes and actions in supporting patients who smoke***

The thematic analysis identified three main themes: role model, lack of motivation of current smokers to help, and counseling/support.

**Role model**

Healthcare professionals expressed mixed feelings about their role as role models. Some acknowledged that their smoking habits set a negative example for patients, while most of them believed that being a former smoker allowed them to serve as a positive influence.

[...] “You are a role model. You can’t smoke like a chimney and tell a patient, ‘Yes, I smoke, but you should quit,’ especially if the patient is healthy and only coming for a check-up”. GP, 40 years old, non-smoker.

[...] “Now I can tell patients with more certainty that they should quit smoking. Before, I hesitated because I was a smoker myself”. RN, 51 years old, former smoker.

Individuals who choose to disclose their smoking status believe that honesty fosters therapeutic communication, which in turn supports patients in deciding to quit smoking.

[...] “If the topic comes up, I mention that I smoke. However, I explain that it’s not right, and although I do it, it’s my mistake. But I still advise them to quit or at least reduce it”. RN, 35 years old, smoker.

[...] “I have never lied about it. Why should I say, ‘No, I don’t smoke,’ when I actually do? We’re in the healthcare field, but I’m not the only smoker”. RN, 40 years old, smoker.

Smoking healthcare professionals reported that when asked by patients if they smoke, they often respond negatively or evade the question, believing they do not set a good example.

[...] “I try to hide my cigarette pack. If it falls out, I feel ashamed”. RN, 56 years old, smoker.

[...] “If asked, I say I don’t smoke. Regardless of my personal beliefs, I must tell patients what medical science says”. GP, 40 years old, smoker.

**Lack of motivation to help patients who smoke**

A prevalent theme was the lack of motivation among healthcare professionals to assist patients with quitting smoking. Indifference in providing support for tobacco treatment was a notable sub-theme.

1. Indifference

Some smoking healthcare professionals reported that their involvement in addressing the smoking behavior of their patients is not their concern. As smokers themselves, they tend to avoid engaging in such discussions.

[...] “I wouldn’t have the inclination or the strength to engage much in trying to persuade them because I would think, ‘Why should I care? I’m a smoker too.’” RN, 55 years old, smoker.

[...] “A nurse who smokes wouldn’t get involved in this matter regarding the patient. They wouldn’t make any comments on it”. RN, 56 years old, smoker.

**Counseling/Support**

In terms of providing support, healthcare professionals varied in their approach. Some placed the responsibility for quitting smoking squarely on the patient, emphasizing personal accountability. Conversely, others highlighted the importance of empathy and understanding in their interactions with patients, recognizing the need for supportive counseling.

[...] “They (the PCPs) know the negative effects of smoking, and that’s why they will advise the patient based on their scientific knowledge that it is not good to smoke and that it is better to quit”. GP, 31 years old, non-smoker.

[...] “I believe I would encourage them more because I know what it means to want to quit and something is holding you back, so I think I would help encourage them”. RN, 41 years old, smoker.

**Empathy**

Some healthcare professionals who are former smokers have greater understanding of the difficulties faced by those trying to quit smoking. They are more supportive, advisory, and guiding.

[...] “I feel that I can talk to them a bit more, and maybe I’m more satisfied now when I tell them they need to quit smoking because I can say that I managed to quit”. GP, 40 years old, former smoker.

[...] “A former smoker who has tried to quit understands the difficulty and can advise and empathize with the patient, the smoker, and can offer tips, so to speak. We all know some general things, but it is different when you have experienced it”. RN, 55 years old, former smoker.

***c***. ***PCPs’ views on the effectiveness of educational programs on smoking cessation***

The analysis of healthcare professionals’ views on the effectiveness of educational programs for tobacco treatment identified three main themes: ineffectiveness, personal decision, and the positive impact of the educational programs.

**Ineffectiveness**

Most of the smokers’ healthcare professionals perceive educational programs as ineffective, offering little benefit to participants. They argue that since they already understand the risks of smoking, these programs have little to offer. Moreover, they view smoking cessation as a personal decision, doubting that such programs can alter someone’s determination if they are not already inclined to quit.

[...] ”I think it won’t affect me much. So, attending an educational program that tells me about the disadvantages of smoking and the consequences, the harmful consequences and so on, as I told you before, we know much better than anyone else”. RN, 46 years old, smoker.

[...] ”I believe it will not help in anything. It’s my issue, so if I want to quit, I will quit, no one else will influence me”. RN, 42 years old, smoker.

**Personal decision**

For some healthcare professionals who smoke, a tobacco treatment educational program may function as supportive, provided that the decision to quit smoking has already been made by the smoker healthcare professional.

[...] ”I really don’t know. It’s how much the other person wants to quit. I think that’s where the help is, regardless of if he is a healthcare professional” GP, 31 years old, smoker.

[...] ”Now how to motivate the healthcare professional himself is a bit difficult, it’s a bit of a decision he must take on his own, I think he knows the risks as a healthcare professional”. GP, 30 years old, smoker.

**Positive Impact**

This theme revealed the sub-theme of the supportive function.

1. Supportive function

A former smoker mentioned that those who smoke and want to quit could find motivation through the educational process of tobacco treatment programs.

[...] “I believe everyone who smokes wants to quit at some point. They seek motivation, and through programs, they can find it”. RN, 51 years old, non-smoker.

A smoker suggested that attending an educational tobacco treatment program could positively influence someone’s decision to quit, as the process might be encouraging.

[...] “A new horizon opens up, and to pass it on to the patient, you might try it yourself. To see if it works. Personally, it might influence me, providing knowledge or tools I could use myself”. RN, 40 years old, smoker.

[..] “They are parallel. And I think if I attended educational... as in other fields, every piece that enters opens another door for you; a window? A balcony door? The roof goes away and you see something else. Another light and gives you another perspective”. RN, 55 years old, smoker.

Several smokers emphasized that attending a tobacco treatment program could support their efforts to quit. They noted that sharing difficulties with others in similar situations within these programs could have a positive impact.

[...] “I think they help. Initially, I wasn’t convinced. Maybe it was too early, or I wasn’t ready, but I think they play a role and help”. RN, 40 years old, smoker.

[...] “It would help. Being around others with the same problem would be beneficial”. RN, 56 years old, smoker.

## 4. Discussion

The aim of this study is to explore the experiences and perspectives of primary care providers regarding their role when delivering tobacco treatment interventions to patients based on their personal smoking status, thereby providing insights that can inform the role of PCP smoking status on clinical practice behavior and the design of more effective interventions to support PCPs with stopping. Understanding the complex factors that influence the delivery of tobacco treatment interventions in primary care is crucial, as these settings represent a vital point of contact for reaching individuals who smoke and providing them with the necessary support to quit [27].

Our qualitative analysis sheds light on the reasons why PCPs smoke, revealing themes that align with and expand upon previous research. Consistent with existing literature, our study identifies stress relief, pleasure, and escapism as primary coping mechanisms for smoking among PCPs [28,29]. Participants described smoking as a vital tool for managing the high levels of stress inherent in their profession, echoing the findings of other studies that highlight the intense pressures faced by healthcare workers [30]. Furthermore, the pleasurable aspects of smoking and its role in providing a momentary escape from daily challenges were prominent in our findings, underscoring the complex emotional and psychological benefits perceived by smokers [31]. Additionally, this study highlighted the theme of addiction, with respondents describing smoking as both a deeply ingrained behavior and a powerful dependency. This mirrors previous research which characterizes smoking among healthcare professionals as a significant addiction issue, compounded by the normalization of smoking within some medical environments [32].

The TPB posits that individual behavior is driven by intentions, which are influenced by attitudes, subjective norms, and perceived behavioral control [33]. In this study, PCPs exhibited internal conflict regarding their smoking habits, which aligns with the TPB’s emphasis on the role of attitudes and perceived norms. Many PCPs felt compelled to conceal their smoking, fearing it would undermine their credibility when advising patients to quit. This reflects a negative attitude towards their behavior, as they recognize the contradiction between their actions and professional responsibilities [34]. Moreover, the findings indicate that some PCPs choose to disclose their smoking status to foster empathy and support among patients. This behavior can be interpreted through the lens of the TPB, where the intention to be transparent is influenced by the belief that sharing personal struggles can enhance patient motivation and trust [35]. The acknowledgment of their smoking habits serves as a means to align their professional role with their personal experiences, thereby potentially improving their effectiveness in counseling patients about cessation. Overall, our findings highlight the complex nature of smoking behaviors among PCPs and suggest that effective tobacco treatment programs should address both the emotional and habitual aspects of smoking.

This investigation revealed multifaceted perspectives among PCPs regarding their role in tobacco treatment delivery, with themes that echo and expand upon prior research. Consistent with other studies, many PCPs feel conflicted about serving as role models for tobacco treatment. While some recognize the importance of setting a positive example, others feel that their current smoking behavior undermines their credibility [28]. The acknowledgment of their influence, particularly among former smokers, aligns with findings that former smokers can leverage their experiences to effectively counsel patients [36]. Additionally, our analysis highlights a significant lack of motivation among smoking PCPs to assist patients in quitting, often due to personal indifference or a perceived lack of responsibility [32]. This lack of motivation is consistent with prior research indicating that PCPs who smoke are less likely to engage in tobacco treatment counseling [28]. Conversely, PCPs who have successfully quit smoking often exhibit greater empathy and a proactive approach towards patient counseling, underscoring the role of personal experience in enhancing the effectiveness of tobacco treatment interventions [36]. COM-B model emphasizes that behavior is a result of the interaction between capability, opportunity, and motivation [37]. In the context of this study, the capability of smoking PCPs to engage in cessation efforts is often undermined by their own smoking habits. Many smoking PCPs feel they do not require additional support in tobacco cessation, relying instead on their personal experiences, which indicates a lack of perceived capability to change their behavior or to effectively counsel patients [38]. In contrast, former smokers among the PCPs demonstrated greater motivation and capability to advocate for cessation, suggesting that their personal success in quitting enhances their role as advocates [39]. Overall, our findings suggest that while PCPs recognize their potential impact on patient smoking behaviors, personal smoking status and motivational barriers significantly influence their engagement in tobacco treatment delivery.

Our study also explored how previous participation in tobacco treatment programs, aimed at improving PCPs’ skills in delivering tobacco treatment to patients, influenced their own smoking behaviors. Thematic analysis revealed that, although smokers PCPs were generally doubtful about the effectiveness of training in changing their smoking behavior, they often found that participating in structured tobacco treatment programs led them to reflect on their own smoking behaviors [13]. The positive outcomes of these educational interventions can be understood through the COM-B model, where training enhances both the capability and motivation of PCPs to engage in smoking cessation efforts. This aligns with previous research indicating that structured training can significantly improve healthcare professionals’ attitudes towards smoking cessation [40]. Moreover, training sessions equipped providers with evidence-based knowledge, practical skills, and motivational strategies that are essential for guiding patients through the cessation process [41,42].

*a*. *Study Limitations*

The results of this study should also be interpreted in light of its limitations. The qualitative nature of the present study has significant strengths in terms of understanding the PCPs’ perspective based on their personal smoking status, however a quantitative evaluation may also be useful in generating data on the frequency of the reported barriers and facilitators for smoking cessation interventions in primary care. Additionally, as with any qualitative research, there is a possibility of researcher bias during data collection, analysis, and interpretation. To mitigate, we employed reflexivity by regularly reflecting on our potential biases, triangulation by involving multiple researchers in data collection and analysis, and peer review to ensure objectivity [43]. This study was conducted in Greece and it is not clear the extent to which study findings would be generalizable to other countries, although it is likely that there would be application to other southern European countries as well as countries with limited cessation supports [3].

## 5. Conclusions

The findings of this study highlight the need for targeted training programs that not only enhance PCPs’ clinical skills but also address their personal smoking behaviors, fostering empathy in tobacco treatment delivery. Additionally, former smokers among healthcare providers can play a pivotal role in counseling patients, emphasizing the importance of peer support. To improve tobacco treatment outcomes in Greece, these findings should be integrated into a primary care setting and tailored to the cultural context, ensuring that PCPs are equipped with the tools and confidence to deliver tobacco treatment to their patients effectively.

In particular, smoking PCPs often experience internal conflict, as they perceive their habits to be at odds with their professional responsibilities. This conflict drives some to conceal their smoking, fearing it compromises their credibility, while others use transparency about their struggles in order to build empathy and motivate patients. However, smoking PCPs are generally less proactive in engaging patients in tobacco treatment efforts, often overestimating the adequacy of their personal experience as a substitute for formal training.

Former smokers among PCPs, conversely, are more motivated and effective in supporting patients, as their personal success reinforces their role as advocates for cessation. Educational programs have demonstrated potential in transforming attitudes and behaviors, with smoking or recently quit PCPs showing greater commitment to tobacco treatment delivery after training. These findings highlight the importance of addressing PCPs’ personal behaviors and attitudes through targeted educational interventions to enhance their effectiveness in promoting smoking cessation among patients.

## Figures and Tables

**Table 1:** Demographic and professional characteristics.

	Profession	Smoking Status	Gender	Age
1	Nurse	Non smoker	Female	54
2	Nurse	Smoker	Female	56
3	Nurse	Smoker	Female	55
4	Physician	Non smoker	Female	31
5	Nurse	Former smoker	Female	51
6	Physician	Smoker	Male	30
7	Nurse	Smoker	Female	46
8	Nurse	Smoker	Female	42
9	Physician	Smoker	Male	66
10	Nurse	Former smoker	Female	55
11	Health Visitor	Former smoker	Female	54
12	Physician	Smoker	Female	65
13	Physician	Former smoker	Female	40
14	Nurse	Smoker	Female	55
15	Nurse	Smoker	Female	40
16	Nurse	Non smoker	Female	52
17	Nurse	Smoker	Female	40
18	Nurse	Smoker	Female	41
19	Physician	Non smoker	Female	40
20	Physician	Smoker	Male	53
21	Nurse	Smoker	Female	35
22	Nurse	Non smoker	Female	55

## Data Availability

The original contributions presented in the study are included in the article. Further inquiries can be directed to the corresponding author.

## Abbreviations

PCP	Primary Care Provider
RN	Registered Nurses
GP	General Practitioners
HV	Health Visitors
